# Comparative safety profiles of risankizumab versus guselkumab: a pharmacovigilance study based on the FAERS database

**DOI:** 10.3389/fphar.2026.1765114

**Published:** 2026-02-18

**Authors:** Shiyi Wang, Yongyi Xu, Hangjuan Lin, Xudong Zhang, Jing Ding, Honggang Jiang, Yihong Fan, Changbo Sun

**Affiliations:** 1 Department of Gastroenterology, Ningbo Municipal Hospital of Traditional Chinese Medicine (TCM), Affiliated Hospital of Zhejiang Chinese Medical University, Ningbo, China; 2 Department of Traditional Chinese Medicine, Ningbo Municipal Hospital of Traditional Chinese Medicine (TCM), Affiliated Hospital of Zhejiang Chinese Medical University, Ningbo, China; 3 Department of Pharmacy, Ningbo Municipal Hospital of Traditional Chinese Medicine (TCM), Affiliated Hospital of Zhejiang Chinese Medical University, Ningbo, China; 4 Department of Gastroenterology, First Affiliated Hospital of Zhejiang Chinese Medical University, Hangzhou, China

**Keywords:** disproportionality analysis, drug safety, FAERS, guselkumab, interleukin-23 inhibitors, pharmacovigilance, risankizumab

## Abstract

**Background:**

Risankizumab and guselkumab are two leading interleukin-23 (IL-23) inhibitors for treating immune-mediated inflammatory diseases. While effective, a direct comparison of their real-world safety profiles is lacking. Understanding the distinct differences in their adverse event (AE) profiles is crucial for clinicians to make appropriate treatment decisions.

**Research Design and Methods:**

We conducted a disproportionality analysis on post-marketing data obtained from the FDA Adverse Event Reporting System (FAERS) database from Q3 2017 to Q2 2025 to detect and compare the AE signals of guselkumab and risankizumab.

**Results:**

A total of 22,470 and 48,141 AE reports for guselkumab and risankizumab, respectively, were analyzed. The analysis revealed markedly different safety profiles. Risankizumab showed significant signals for skin cancer (particularly in patients ≥65 years), intestinal obstruction, and other newly identified serious events such as myocardial infarction and cerebrovascular accident. In contrast, guselkumab’s primary signals were dominated by medication management issues like “product dose omission issue” and “accidental exposure to product,” which correlated with reports of decreased therapeutic effect. Unique, unlabeled signals for guselkumab included a different spectrum of AEs, such as Hodgkin’s disease, pemphigoid, and autoimmune thyroiditis. Furthermore, the median time-to-onset was significantly shorter for guselkumab (62 days) compared to risankizumab (168 days).

**Conclusion:**

Guselkumab and risankizumab exhibit divergent real-world safety profiles, challenging the notion of a uniform class effect. Clinicians should be vigilant for malignancies and serious systemic events with risankizumab, particularly in the elderly, while prioritizing patient education for guselkumab to prevent administration errors. These findings support individualized treatment strategies to minimize drug-specific risks.

## 1 Introduction

Interleukin-23 (IL-23) plays a pivotal role in the development and function of T helper cells, with Th17 cells emerging as a significant T cell subset involved in the pathogenesis of immune-mediated diseases (Sharma et al., 2022). IL-23 is a heterodimeric cytokine composed of the IL-23-specific p19 subunit (IL-23p19) and the p40 subunit, which is shared with IL-12 (IL-23p40). Its primary sources include myeloid cells such as monocytes, macrophages, and dendritic cells (Krueger et al., 2024). Studies have shown that elevated levels of IL-23 are detectable in the affected skin of patients with psoriasis (PsO) (Kelemen et al., 2022), synovial tissue in patients with psoriatic arthritis (PsA) (Hum et al., 2025), and intestinal mucosa in patients with Crohn’s disease (CD) (Liu et al., 2011).

As a result, selective blockade of the IL-23 pathway has become an effective therapeutic strategy for these inflammatory diseases (Ghoreschi et al., 2021; García-Domínguez, 2025). Therapeutic monoclonal antibodies targeting the IL-23p19 subunit, such as guselkumab and risankizumab, have demonstrated efficacy in a variety of conditions including PsO (Yang et al., 2021), PsA (Mohanakrishnan et al., 2022), CD (Fanizza et al., 2023), and ulcerative colitis (UC) (Saab et al., 2025). Risankizumab (brand name SKYRIZI®) was approved by the U.S. FDA in April 2019 for the treatment of moderate-to-severe PsO and was later approved in January 2022 and June 2022 for active PsA and moderate-to-severe active CD (Pang et al., 2024), respectively. In June 2024, it was further approved for the treatment of moderate-to-severe active UC in adult patients (Johnson and Loftus, 2024). Guselkumab (brand name TREMFYA®) has also received similar indications (US Food and Drug Administration, 2025), and these two drugs have become representative therapies within the IL-23 inhibitor class.

Although risankizumab and guselkumab share the same core mechanism of action (Bourgonje et al., 2025), which involves binding specifically to the IL-23p19 subunit to block its interaction with the receptor, they possess distinct molecular structures. Guselkumab is a fully human IgG1λ monoclonal antibody with a natural Fc region, whereas risankizumab’s Fc region is engineered with L234A and L235A mutations (known as the “LALA” substitution) (Cross, 2025). This modification significantly weakens its ability to bind to Fcγ receptors (such as CD64). A series of *in vitro* studies have shown that guselkumab, with its natural Fc region, can bind to CD64^+^ myeloid cells (the primary source of IL-23 in PsA) and capture IL-23 secreted by these cells. This dual mechanism of action has been described as a potential “bifunctional” effect (Doggrell, 2025). In contrast, the “LALA” modification of risankizumab precludes this function. Further experiments have demonstrated that guselkumab exhibits enhanced Fc-dependent efficacy in inhibiting IL-23 signaling (Sachen et al., 2025). However, the long-term clinical implications of these *in vitro* findings remain uncertain, and further research is required to determine whether these molecular differences have a practical impact on clinical outcomes, including efficacy and safety.

To date, while the safety profiles of IL-23 inhibitors relative to older biologics (e.g., tumor necrosis factor-α inhibitors) have been studied, there remains a lack of direct comparative pharmacovigilance analyses specifically between risankizumab and guselkumab in real-world practice. Given their structural differences, distinguishing their specific safety signals is clinically relevant. Therefore, pharmacovigilance analysis using large-scale real-world data is crucial for identifying and comparing the safety profiles of these two drugs across diverse populations. This study aims to conduct a comprehensive comparative analysis of post-marketing adverse event (AE) reports for risankizumab and guselkumab using the FDA Adverse Event Reporting System (FAERS) database, providing valuable, data-driven evidence to guide therapeutic choices for patients with immune-mediated inflammatory diseases.

## 2 Methods

### 2.1 Data source

This is a retrospective observational pharmacovigilance study utilizing the FAERS database, which serves as a global source for post-marketing AE reports (Sakaeda et al., 2013). The FAERS database is publicly available and contains AE reports submitted by healthcare professionals, pharmaceutical manufacturers, and consumers. Reports are updated quarterly and include seven datasets: patient demographics and management data, drug information, AE details, patient outcomes, report sources, drug treatment start and end dates, and drug indications. For this study, data from the period between Q3 2017 and Q2 2025 were retrieved, taking into account the marketing authorization dates of the drugs under investigation.

### 2.2 Data extraction and analysis

Duplicate reports were removed. For data entries in the DEMO table with the same caseid, only the latest report based on date was retained. The relationship between datasets was established using the primaryid field, and any abnormalities in age and weight metrics were corrected. Drug names were standardized using the Medex_UIMA_1.8.3 system. The focus of this study was on two IL-23 inhibitors: guselkumab and risankizumab. To ensure comparability, this study excludes tildrakizumab, which is only indicated for PsO (Reich et al., 2017a), and mirikizumab, which was approved in October 2023 and has insufficient data accumulation (Keam, 2023). For comprehensive drug identification, both generic and brand names were used, with the following keywords: “Guselkumab/TREMFYA/” and “Risankizumab/SKYRIZI/ABBV-066”. Only reports identifying the drug as the primary suspect were retained. All data were downloaded from the FDA website (https://fis.fda.gov/extensions/FPD-QDE-FAERS/FPD-QDE-FAERS.html) and imported into R 4.5.1 for data cleaning and analysis.

We collected clinical characteristics of patients with AEs associated with guselkumab and risankizumab, including sex, age, reporting region, report time, and outcomes. Table 1 describes the characteristics of unique cases after deduplication, where multiple events within a single report were aggregated at the case level for demographic summaries. Severe AE outcomes were defined as hospitalization, disability, life-threatening conditions, or death.

**TABLE 1:** Characteristics of reports associated with Risankizumab and Guselkumab from Q3 of 2017 to Q2 of 2025.

Factors	Risankizumab	Guselkumab	Total
Number of reports (cases)	48141	22470	70611
Number of adverse events (AEs)	112265	37380	149645
Gender			
Female	25553 (53.08)	10785 (48.00)	36338 (51.46)
Male	19906 (41.35)	7857 (34.97)	27763 (39.32)
Unknown	2682 (5.57)	3828 (17.04)	6510 (9.22)
Age	59.00 (46.00,69.00)	53.00 (42.00,62.00)	​
<18	50 (0.10)	25 (0.11)	75 (0.11)
18–65	13790 (28.65)	9634 (42.87)	23424 (33.17)
≥65	7831 (16.27)	2292 (10.20)	10123 (14.34)
Unknow	26470 (54.98)	10519 (46.81)	36989 (52.38)
Reporter			
Consumer	39417 (81.88)	8355 (37.18)	47772 (67.66)
Pharmacist	3823 (7.94)	9458 (42.09)	13281 (18.81)
Physician	3357 (6.97)	3406 (15.16)	6763 (9.58)
Unknown	1501 (3.12)	709 (3.16)	2210 (3.13)
Other health-professional	38 (0.08)	537 (2.39)	575 (0.81)
Lawyer	5 (0.01)	5 (0.02)	10 (0.01)
Serious outcomes			
Death	2080 (6.25)	324 (5.71)	2404 (3.40)
Disability	182 (0.55)	50 (0.88)	232 (0.33)
Life threatening	173 (0.52)	132 (2.33)	305 (0.43)
Hospitalization	10784 (32.38)	1530 (26.97)	12314 (17.44)
Other serious	19984 (60.01)	3601 (63.49)	23585 (33.40)
Reported countries			
United States	38298 (79.55)	19128 (85.13)	57426 (81.33)
Canada	3329 (6.92)	776 (3.45)	4105 (5.81)
United Kingdom	1121 (2.33)	310 (1.38)	1431 (2.03)
Germany	644 (1.34)	394 (1.75)	1038 (1.47)
Other countries	2976 (6.18)	675 (3.00)	3651 (5.17)

### 2.3 Statistical analysis

In our study, disproportionality measures commonly used in pharmacovigilance studies were employed to identify potential signals between drugs and AEs (Montastruc et al., 2011). Disproportionality analysis is a widely used data mining method that compares the frequency ratios of AEs between the exposed and unexposed populations using a 2x2 contingency table (Noguchi et al., 2021) (Supplementary Table S1). In this study, the proportional reporting ratio (PRR), reporting odds ratio (ROR), and Bayesian confidence propagation neural network (BCPNN) algorithms were applied. The equations and standards for these three algorithms are listed in Supplementary Table S2. The use of multiple algorithms allows for cross-validation to reduce the generation of false positive signals (Chen et al., 2008). In our study, only signals with at least three AE records related to the target drugs were considered. A positive signal for drug-related AEs was defined as meeting the following criteria for at least one of the algorithms: lower limit of 95% confidence interval (CI) > 1, N ≥ 3; PRR ≥2, χ^2^ ≥ 4, N ≥ 3; IC025 > 0 (Zou et al., 2024). Subgroup analysis was performed based on age groups (<18 years, 18–65 years, and >65 years), as the AE risk across different age groups is not well understood. Additionally, time-to-onset patterns were evaluated using Kaplan-Meier cumulative incidence curves and Log-rank tests, with Cox proportional hazards regression used to estimate Hazard Ratios (HRs) and 95% CIs. Statistical analyses were performed using R4.5.1. A higher value indicates stronger signal intensity, suggesting a stronger association between the target drug and the AE.

### 2.4 Signal filtering and classification

The latest version of the Medical Dictionary for Regulatory Activities (MedDRA 25.0) was used to match the preferred terms (PT) and system organ classes (SOC) for AEs associated with guselkumab and risankizumab. These were used for signal coding, classification, and localization to analyze the specific SOCs involved in AE signals. PTs related to indications or associated complications were excluded to prevent misclassification.

## 3 Results

### 3.1 Descriptive analyses

**FIGURE 1:**
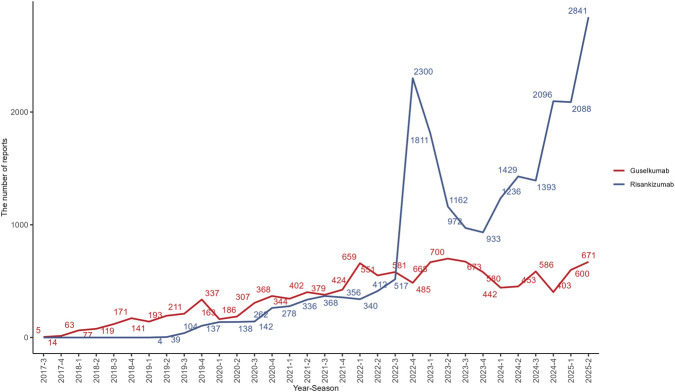
Quarterly adverse event reports for risankizumab (blue line) and guselkumab (red line) following their market approval.

From Q3 2017 to Q2 2025, a total of 11,090,223 AE reports were collected in the FAERS database, with 48,141 reports related to risankizumab and 22,470 related to guselkumab. The clinical characteristics of the patients are presented in Table 1. Guselkumab was approved for use in July 2017, and the number of reports showed a continuous and steady increase, peaking at 1,488 reports in Q2 2025, the final time point of this study. In contrast, risankizumab was approved for use in April 2019, and after a gradual increase in reports in the early stages, it saw a significant peak of 7,976 reports in Q4 2022, as shown in Figure 1. The majority of reports came from the United States, followed by Canada, the United Kingdom, and Germany, among other countries.

The majority of reports for risankizumab came from consumers (81.88%), while reports for guselkumab were primarily from pharmacists (42.09%) and consumers (37.18%). For both drugs, AE reports for females slightly outnumbered those for males. In terms of serious outcomes, risankizumab showed a higher proportion of reports for hospitalization (32.38% vs. 26.97%) and death (6.25% vs. 5.17%), while guselkumab had a significantly higher proportion of life-threatening events compared to risankizumab (2.33% vs. 0.52%).

We summarized the top three indications and the top five concomitant medications based on the total number of AE reports for both drugs (Table 2). The primary indication for both risankizumab and guselkumab was PsO, accounting for 60.74% and 41.04% of reports, respectively. For Risankizumab, the second most common indication was CD (18.7%), far exceeding PsA (6.67%). For guselkumab, PsA (9.68%) and UC (1.00%) ranked second and third, respectively.

**TABLE 2:** Top three indications and top five concomitant drugs in adverse events reports of risankizumab and guselkumab.

Category	Risankizumab (n, %)	Guselkumab (n, %)
Indications	PsO (29292, 60.74)	PsO (9068, 41.04)
	CD (9017, 18.7)	PsA (2139, 9.68)
	PsA (3218, 6.67)	UC (221, 1.00)
Concomitant medication	Atorvastatin (852, 2.09)	Metformin (204, 2.34)
	Aspirin (760, 1.86)	Lisinopril (162, 1.86)
	Metformin (698, 1.71)	Amlodipine (161, 1.85)
	Omeprazole (646, 1.58)	Aspirin (157, 1.81)
	Amlodipine (645, 1.58)	Atorvastatin (152, 1.75)

Abbreviations: CD, Crohn’s disease; PsA, psoriatic arthritis; PsO, psoriasis; UC, ulcerative colitis.

In the AE reports for risankizumab, the most common concomitant medication was atorvastatin (2.09%), followed by aspirin (1.86%) and metformin (1.71%). Omeprazole and amlodipine ranked fourth and fifth, each accounting for approximately 1.58%. In contrast, for guselkumab, metformin was the most commonly reported concomitant medication (2.34%), being the only drug with a proportion exceeding 2%. It was followed by lisinopril (1.86%) and amlodipine (1.85%). Aspirin (1.81%) and atorvastatin (1.75%) ranked as the last two in the list of top concomitant medications.

### 3.2 Disproportionality analyses

#### 3.2.1 SOC-level AE analysis

**FIGURE 2:**
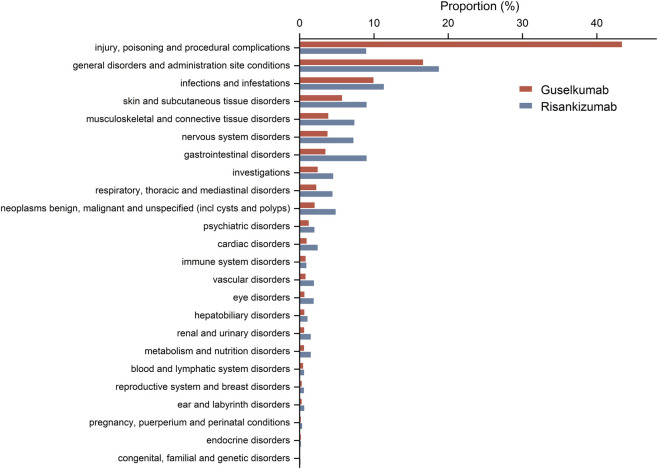
Proportional distribution of reported adverse events by system organ class for risankizumab (blue bars) and guselkumab (red bars).

A total of 24 system organ classes (SOCs) were involved in the AEs related to guselkumab and risankizumab, with the distribution of reported cases across these SOCs shown in Figure 2. The most commonly reported SOCs for guselkumab were Injury, poisoning and procedural complications, General disorders and administration site conditions, and Infections and infestations. For risankizumab, the most commonly reported SOCs were General disorders and administration site conditions, Infections and infestations, and Skin and subcutaneous tissue disorders.

#### 3.2.2 Comparison of safety signals in four SOCs

**FIGURE 3:**
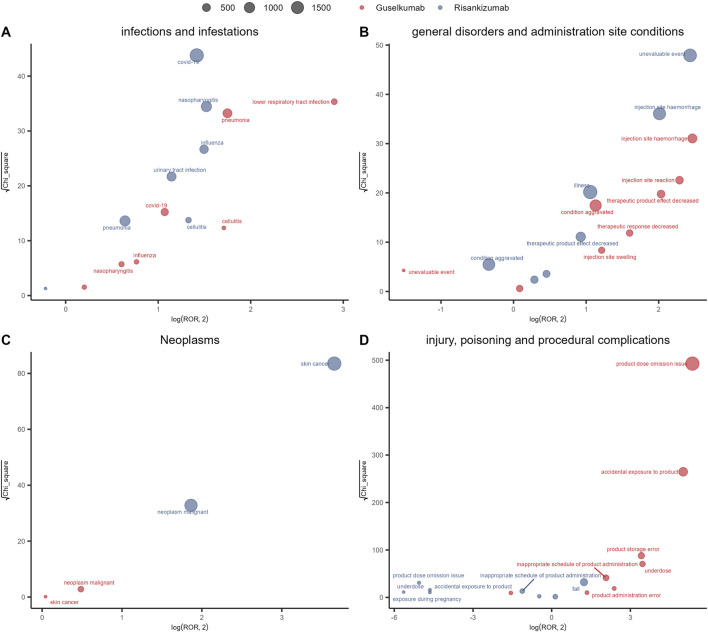
Signal detection analysis of adverse events for risankizumab (blue points) and guselkumab (red points) within four key system organ classes (SOCs): **(A)** Infections and infestations, **(B)** General disorders and administration site conditions, **(C)** Neoplasms, and **(D)** Injury, poisoning and procedural complications. Each point represents a specific adverse event, with its size proportional to the number of reports. The x-axis plots the log-base-2 of the Reporting Odds Ratio (log_2_ (ROR)), while the y-axis plots the square root of the chi-square value (√χ^2^). In these plots, signals in the upper-right quadrant represent events with both a high reporting ratio and strong statistical significance.

We compared the AE signals across four SOCs and observed distinct characteristics in the signals for guselkumab and risankizumab, as shown in Figure 3. In the Infections and infestations category, the most prominent signal for risankizumab was COVID-19, with both a high chi-square value (Y-axis) and a large number of reports (bubble size). In contrast, guselkumab’s strongest signals in this category (measured by the reporting odds ratio, or ROR) were low respiratory tract infection and tuberculosis, which showed a higher ROR.

In the General disorders and administration site conditions category, guselkumab’s most significant safety signal was injection site hemorrhage, accompanied by several high-risk signals related to “reduced efficacy.” In contrast, risankizumab’s major signals pointed to hemia and unevaluable event. In the Neoplasms category, the primary and most significant signals for risankizumab were skin cancer, basal cell carcinoma, and malignant neoplasm. By comparison, guselkumab’s signals in this category were very weak. In the Injury, poisoning and procedural complications category, product dose omission issue was the predominant signal for guselkumab, while risankizumab showed an ROR <0 for this issue, indicating that its report frequency was much lower than expected.

#### 3.2.3 PT-level AE analysis

Since the FAERS database includes all PTs related to healthcare and medicine, it also captures signals unrelated to the drugs, which may be caused by disease progression, medication errors, or product quality issues. After excluding drug-unrelated signals, a total of 151 and 123 signals were identified for risankizumab and guselkumab, respectively. Detailed summaries of these positive PTs (ranked by report frequency) are provided in Supplementary Tables S3, 4.

The top five strongest signals for risankizumab were injection site papule, *mycobacterium tuberculosis* complex test positive, intestinal stenosis, skin plaque, and obstruction (Table 3). The top five signals by report frequency were pruritus, COVID-19, arthralgia, fall, and nasopharyngitis (Supplementary Table S3). Additionally, 39 previously unreported signals were identified, including notable signals related to malignant tumors (e.g., skin cancer, malignant melanoma, and colon cancer), severe cardiovascular and cerebrovascular events (e.g., myocardial infarction, cerebrovascular accident, and pulmonary thrombosis), and life-threatening gastrointestinal events (e.g., intestinal obstruction, intestinal perforation, and pancreatitis).

**TABLE 3:** Top 20 signal strength of adverse events of risankizumab at the preferred terms level in FAERS database.

Preferred terms	N	ROR (95% CI)	PRR (95% CI)	chisq	IC(IC025)
Injection site papule	164	26.73 (22.73, 31.42)	26.69 (22.82, 31.22)	3627.78	4.58 (4.35)
*mycobacterium tuberculosis* complex test positive	77	17.45 (13.84, 22.01)	17.44 (13.78, 22.06)	1108.18	4.02 (3.69)
Intestinal stenosis	78	16.46 (13.08, 20.72)	16.45 (13, 20.81)	1055.34	3.95 (3.62)
Skin plaque	233	16.29 (14.26, 18.6)	16.25 (14.17, 18.64)	3112.78	3.93 (3.74)
Obstruction	139	15.32 (12.9, 18.2)	15.31 (12.83, 18.26)	1741.24	3.85 (3.6)
Skin cancer	692	12.62 (11.69, 13.63)	12.55 (11.6, 13.57)	6975.21	3.58 (3.47)
Skin disorder	751	12.41 (11.53, 13.36)	12.33 (11.4, 13.34)	7421.76	3.55 (3.45)
Intestinal obstruction	652	10.33 (9.55, 11.18)	10.28 (9.5, 11.12)	5225.33	3.3 (3.19)
Small intestinal obstruction	188	10.31 (8.9, 11.93)	10.29 (8.97, 11.8)	1508.77	3.31 (3.1)
Coronary artery occlusion	139	10.18 (8.59, 12.06)	10.17 (8.53, 12.13)	1099.64	3.29 (3.04)
Basal cell carcinoma	285	9.9 (8.79, 11.14)	9.87 (8.77, 11.1)	2178.68	3.25 (3.08)
Melanocytic naevus	79	9.74 (7.77, 12.2)	9.73 (7.84, 12.07)	593.41	3.23 (2.91)
Meniscus injury	107	9.28 (7.65, 11.26)	9.28 (7.63, 11.29)	759.05	3.16 (2.88)
Rotator cuff syndrome	177	9.24 (7.95, 10.74)	9.22 (7.88, 10.79)	1247.18	3.15 (2.94)
Gastrointestinal sounds abnormal	74	8.94 (7.08, 11.27)	8.93 (7.06, 11.3)	501.41	3.11 (2.78)
Arterial occlusive disease	84	8.85 (7.12, 11.01)	8.85 (7.13, 10.98)	562.64	3.1 (2.78)
Hernia	295	8.26 (7.35, 9.28)	8.24 (7.33, 9.27)	1812.07	3 (2.83)
Post procedural infection	130	8.2 (6.88, 9.77)	8.19 (6.87, 9.77)	792.21	2.99 (2.74)
Gastrointestinal inflammation	107	7.79 (6.42, 9.45)	7.78 (6.4, 9.46)	611.64	2.92 (2.64)
Squamous cell carcinoma of skin	97	7.78 (6.35, 9.52)	7.77 (6.39, 9.45)	553.23	2.92 (2.63)

For guselkumab, the top five strongest signals were product dose omission issue, viral pericarditis, accidental exposure to product, parapsoriasis, and injection site plaque (Table 4). The top five signals by report frequency were product dose omission issue, accidental exposure to product, product storage error, inappropriate schedule of product administration, and pneumonia (Supplementary Table S4). Additionally, 49 previously unrecorded signals were identified, with notable new signals related to malignant tumors (e.g., lung adenocarcinoma, malignant melanoma, and B-cell lymphoma), severe autoimmune skin diseases (e.g., pemphigoid and exfoliative dermatitis), and rare but serious cardiovascular and neurological events (e.g., dilated cardiomyopathy and transverse myelitis).

**TABLE 4:** Top 20 signal strength of adverse events of guselkumab at the preferred terms level in FAERS database.

Preferred terms	N	ROR (95% CI)	PRR (95% CI)	chisq	IC(IC025)
Product dose omission issue	8242	41.23 (40.22, 42.27)	32.36 (31.73, 33)	242939.36	4.96 (4.93)
Viral pericarditis	3	35.66 (11.23, 113.22)	35.66 (11.22, 113.34)	96.96	5.1 (3.65)
Accidental exposure to product	2569	32.33 (31.04, 33.68)	30.18 (29.02, 31.39)	70137.22	4.87 (4.81)
Parapsoriasis	4	29.88 (11.02, 80.99)	29.87 (10.99, 81.16)	107.81	4.85 (3.56)
Injection site plaque	4	25.77 (9.53, 69.7)	25.77 (9.48, 70.02)	92.41	4.65 (3.36)
*mycobacterium tuberculosis* complex test positive	35	20.94 (14.97, 29.28)	20.92 (14.99, 29.19)	647.78	4.35 (3.88)
Eczema nummular	5	20.1 (8.28, 48.79)	20.09 (8.32, 48.53)	88.6	4.3 (3.13)
Nail pitting	3	17.58 (5.6, 55.16)	17.58 (5.64, 54.79)	45.96	4.11 (2.68)
Tracheomalacia	4	16.8 (6.24, 45.19)	16.79 (6.3, 44.74)	58.26	4.04 (2.76)
Latent tuberculosis	22	14.92 (9.79, 22.75)	14.91 (9.69, 22.95)	280.6	3.87 (3.28)
Fibroadenoma of breast	3	14.81 (4.73, 46.37)	14.81 (4.75, 46.16)	37.95	3.86 (2.43)
angioimmunoblastic t-cell lymphoma	3	13.84 (4.42, 43.3)	13.83 (4.44, 43.11)	35.14	3.77 (2.34)
Spontaneous bacterial peritonitis	3	12.41 (3.97, 38.8)	12.41 (3.98, 38.68)	31.02	3.61 (2.19)
Administration site reaction	3	12.11 (3.88, 37.87)	12.11 (3.89, 37.74)	30.16	3.58 (2.15)
Renal cancer metastatic	4	11.89 (4.43, 31.89)	11.89 (4.46, 31.68)	39.33	3.55 (2.28)
Antinuclear antibody increased	5	11.37 (4.71, 27.49)	11.37 (4.71, 27.47)	46.68	3.49 (2.33)
Underdose	560	11.03 (10.14, 11.99)	10.88 (10.06, 11.77)	4966.43	3.43 (3.31)
Nasopharyngeal cancer	3	10.73 (3.44, 33.51)	10.73 (3.44, 33.44)	26.13	3.41 (1.98)
Product storage error	912	10.71 (10.02, 11.44)	10.47 (9.87, 11.1)	7736.23	3.37 (3.28)
Dactylitis	5	10.55 (4.37, 25.49)	10.55 (4.37, 25.49)	42.68	3.38 (2.22)

### 3.3 Time-to-onset analysis

**FIGURE 4:**
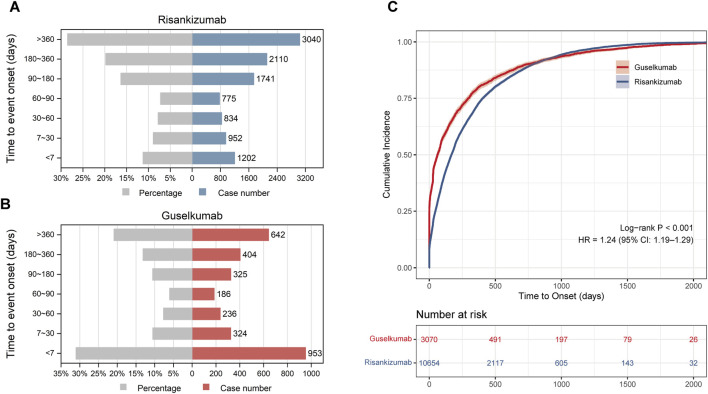
Time-to-onset distribution and cumulative incidence of adverse events associated with **(A)** risankizumab and **(B)** guselkumab. Panels **(A)** and **(B)** display the distribution of reports across time intervals, showing both the percentage of reports (grey bars) and the absolute number of cases (blue bars for risankizumab, red bars for guselkumab). **(C)** Cumulative incidence curves comparing the time-to-onset of adverse events. The red line (guselkumab) and blue line (risankizumab) indicate the cumulative probability of event onset over time. The difference between groups was statistically significant (Log-rank test, *P* < 0.001), with a Hazard Ratio (HR) of 1.24 (95% CI: 1.19–1.29) derived from the Cox proportional hazards model.

A time-to-onset analysis was conducted to examine the timing patterns of AEs associated with IL-23 inhibitors. After excluding reports with missing or incorrect onset dates, a total of 13,724 reports were included: 10,654 for risankizumab and 3,070 for guselkumab. The time-to-onset patterns for AEs with both drugs showed significant differences (Log-rank test, P < 0.001; Figure 4). Cox proportional hazards regression analysis further quantified this disparity, yielding a Hazard Ratio (HR) of 1.24 (95% CI: 1.19–1.29), indicating that guselkumab is associated with a significantly earlier onset of AEs compared to risankizumab.

Consistent with these findings, the median time-to-onset for risankizumab was longer, at 168.00 days (IQR: 47.00, 405.00 days), compared to 62 days (IQR: 1.00, 295.00 days) for guselkumab. This difference was particularly pronounced in the early stages of treatment. Within the first 30 days of treatment, 1,277 AE reports were received for guselkumab, accounting for 41.60% of the total, with 31.04% of those occurring in the first week. In contrast, AE reports for risankizumab within the first 30 days accounted for a lower proportion of its total reports, at 20.22% (2,154 reports).

To further quantify the risk patterns, a Weibull-shape parameter (WSP) analysis was conducted. The results showed that the shape parameters (β values) for both drugs were significantly less than 1 (risankizumab: 0.69; guselkumab: 0.44), indicating early failure-type patterns. This suggests that the risk of AEs for both drugs is highest in the initial stages of treatment (Table 5).

**TABLE 5:** Weibull shape parameter test for adverse events associated with risankizumab and guselkumab.

Drugs	Available *N*	Median (IQR)	Weibull distribution		Failure patterns
			α (95%CI)	β (95% CI)	
Risankizumab	10,654	168 (47, 405)	241.61 (234.77, 248.66)	0.69 (0.68, 0.70)	Early failure
Guselkumab	3,070	62 (1, 295)	109.71 (100.87, 119.33)	0.44 (0.43, 0.46)	Early failure

### 3.4 Subgroup analysis

**FIGURE 5:**
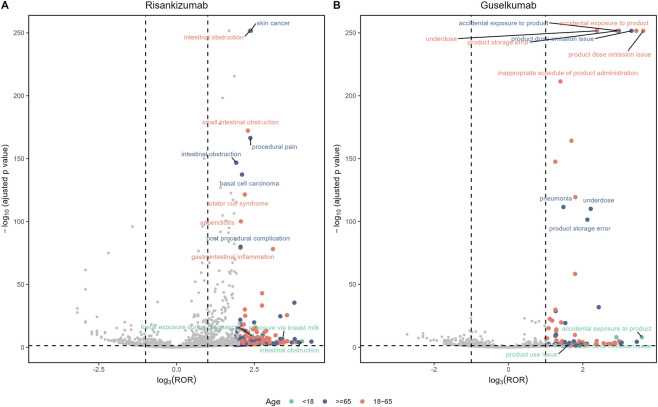
Volcano plots comparing adverse event (AE) signals for **(A)** risankizumab and **(B)** guselkumab, stratified by patient age. The x-axis represents signal strength (log_3_ (ROR)), and the y-axis represents statistical significance (-log_10_ adjusted p-value). Each point is an AE, color-coded by age group: <18 years (teal), 18–65 years (red), and ≥65 years (blue). In these plots, signals in the upper-right quadrant represent AEs with both high statistical significance and a strong reporting ratio. The most prominent signals are labeled by name.

Age-based subgroup analysis revealed differences in the AE signal profiles between risankizumab and guselkumab, particularly among elderly patients (Figure 5). The analysis indicated that for individuals aged 65 and older, the risk of skin cancer (ROR = 13.69, IC = 3.70) and basal cell carcinoma (ROR = 10.09, IC = 3.28) was higher for risankizumab. Notably, the intestinal obstruction signal was highly significant in both the ≥65 years and 18–65 years age groups. In contrast, guselkumab’s signal profile was primarily composed of errors or issues related to product use and management, such as accidental exposure to product and product dose omission issue.

## 4 Discussion

This study, based on real-world data from the FAERS database, presents the first comprehensive and systematic comparative analysis of the AE profiles of two important IL-23 inhibitors: guselkumab and risankizumab. Our findings reveal significant differences between the two drugs in terms of baseline characteristics, indication distribution, AE signal categories, key risk signals, and event timing patterns, providing new evidence for clinical safety monitoring and risk management.

A core finding of this study is that the primary reported events for guselkumab were not pharmacologically related AEs, but rather a series of drug administration and management-related issues, such as product dose omission and product storage error. This phenomenon is strongly linked to its primary indications (e.g., PsO), a chronic disease requiring long-term self-injection by patients. These management-related events coexist with high-frequency reports of therapeutic product effect decreased. This contrasts with controlled clinical settings like the VOYAGE one trial, where strict protocols minimized administration errors, though mild injection site reactions were still reported (Blauvelt et al., 2017). Therefore, our findings suggest that in a real-world setting, improper drug administration and poor adherence may be key factors contributing to suboptimal clinical outcomes, rather than a decrease in the drug’s efficacy. This contrasts with risankizumab, which primarily presented signals linked to events with clear pathophysiological significance. To address the interference from administration-related events for guselkumab, we stratified our analysis by specific SOCs (e.g., neoplasms and infections) to specifically compare the pharmacological safety profiles of the two agents.

Regarding cancer risk, the disproportionality analysis revealed notable signal differentiation. Risankizumab showed strong associations with non-melanoma skin cancers (NMSC) (Fahradyan et al., 2017), particularly basal cell carcinoma and squamous cell carcinoma of the skin, especially in patients aged 65 and older. This aligns with safety data from pivotal trials in PsO (UltIMMa-1/2) and UC (COMMAND), which also reported NMSC and other malignancies, though these events remained rare and comparable to placebo (Gordon et al., 2018; Louis et al., 2024). While PsO patients inherently have a higher baseline risk for NMSC (Wang et al., 2020) and existing clinical trial data have not confirmed an increased risk (Gordon et al., 2024), the signal in the FAERS database, notable for both its high reporting frequency and strong statistical disproportionality, suggests this potential association with NMSC warrants attention in long-term post-market surveillance. In contrast, guselkumab did not show significant skin cancer signals, but ROR analysis identified a rare signal for angioimmunoblastic T-cell lymphoma, a hematologic malignancy (ROR = 13.84, IC = 3.77). Notably, our FAERS findings of specific malignancies align with observations from the VOYAGE 1/2 (PsO) and QUASAR (UC) trials, which documented sporadic cases of basal cell carcinoma, prostate cancer, and breast cancer in treatment arms (Blauvelt et al., 2017; Reich et al., 2017b; Peyrin-Biroulet et al., 2023). This consistency between trial case reports and real-world signals suggests that, while the absolute risk remains low, vigilance regarding malignancy is warranted. The differential signals imply that the potential carcinogenic risks of the two drugs may involve different targets or mechanisms, which warranting further clarification through future epidemiological studies.

Regarding infection risk, both drugs exhibited signals consistent with the known class effects of biologics (Ouranos et al., 2025). Both drugs showed associations with tuberculosis. Notably, risankizumab displayed a particularly strong signal for *mycobacterium tuberculosis* complex test positive (ROR = 17.45, IC = 4.02), while guselkumab was associated with latent tuberculosis. However, triangulation with Phase 3 trials suggests that these signals may reflect screening bias rather than true toxicity. In particular, serious infection rates in the GALAXI (CD) and QUASAR (UC) studies were comparable to or even lower than placebo, likely due to better disease control (Peyrin-Biroulet et al., 2023; Panaccione et al., 2025). This highlights the importance of rigorous tuberculosis screening before initiating IL-23 inhibitor therapy. Although retrospective studies have shown that IL-23 inhibitors do not increase the risk of reactivation of asymptomatic latent tuberculosis infection (LTBI) (Torres et al., 2024), a recent population-based TriNetX analysis indicated a higher incidence of tuberculosis in patients treated with IL-23 inhibitors compared to the general population (Woodie et al., 2025). Additionally, COVID-19 was a high-frequency reported event for both drugs, but the signal strength (ROR) was not significant, suggesting that the signal likely reflects the global pandemic context during the analysis period rather than a significant drug-specific risk increase.

This study also identified and summarized potential new safety signals beyond the drug labels. For risankizumab, we found potential associations with various serious cardiovascular and cerebrovascular events (e.g., cerebrovascular accident, myocardial infarction), gastrointestinal events (e.g., intestinal obstruction), and malignancies (e.g., skin cancer, colon cancer). Although early Phase 2 trials did not report major cardiovascular events (Feagan et al., 2018), recent case reports (Polat Ekinci et al., 2025) have shown that two PsO patients developed cerebrovascular accidents following risankizumab treatment, and other FAERS analyses have suggested a potential increase in cardiovascular event risk (Egeberg and Thyssen, 2023; Woods, 2023). Given that such trials are often underpowered to detect rare events, further long-term observational data are needed. For guselkumab, newly identified safety signals mainly focused on a broader category of malignancies (e.g., Hodgkin’s disease, lung adenocarcinoma), severe skin reactions (e.g., pemphigoid, dermatitis exfoliative generalised), and rare autoimmune diseases (e.g., autoimmune thyroiditis, myelitis transverse). These real-world signals are consistent with rare observations from the VOYAGE one and two trials, which documented sporadic cases of malignancies and immune-mediated reactions in treatment arms (Blauvelt et al., 2017; Reich et al., 2017b). Future studies should further validate these new AE signals to elucidate their clinical significance and inform intervention strategies.

The differences in safety signal profiles suggest that the mechanisms of action of the two drugs may not be identical. A key molecular distinction lies in their Fc fragment function. Guselkumab is a fully human IgG1λ antibody with a natural Fc region. Studies have shown that its Fc region can bind to CD64 (FcγRI) on myeloid cells (such as macrophages) (Pugliano et al., 2025), potentially triggering broader immune regulatory effects beyond simple IL-23 blockade. This may explain the more diverse autoimmune and skin-related safety signals observed with guselkumab. In contrast, risankizumab is a humanized IgG1 antibody, with an engineered Fc region (e.g., L234A and L235A mutations) designed to significantly reduce binding to Fcγ receptors (including CD64) and complement C1q, minimizing unnecessary cytotoxic effects. This “silencing” difference in molecular design likely accounts for the distinct safety profiles of the two drugs in real-world clinical use. Our findings emphasize that even monoclonal antibodies targeting the same cytokine, subtle structural differences (particularly in the Fc region) can translate into clinically significant safety differences, which are crucial for guiding clinical decision-making and risk monitoring.

Finally, the time-to-onset analysis, reinforced by survival analysis models, revealed distinct temporal risk profiles for the two drugs. The Cox regression analysis (HR = 1.24) and cumulative incidence curves confirmed that AEs for guselkumab were more likely to occur early in treatment (especially within the first 30 days), suggesting that early risk management (such as patient education and post-injection observation) is particularly important. In contrast, risankizumab’s AE time-to-onset had a longer median, with a relatively gradual and delayed risk profile, indicating that more prolonged monitoring is needed for patients using risankizumab.

This study has several inherent limitations. First, as FAERS is a spontaneous reporting system, it is subject to reporting bias (e.g., selective reporting, underreporting, or duplicate reporting) and data gaps (e.g., incomplete information on clinical details or disease severity). Second, the disproportionality analysis used in this study can only identify signal associations and cannot establish causality. Third, the large disparity in report numbers (particularly the surge in risankizumab reports in 2022) may affect the stability of signal detection. Fourth, the data in this study were predominantly from the United States (risankizumab: 79.55%; guselkumab: 85.13%). As a result, the findings may reflect specific U.S. clinical practices and patient demographics, which could limit the generalizability of the safety profiles to populations outside the United States. Finally, the FAERS database lacks comprehensive clinical data, specifically detailed prior disease history. While our analysis of concomitant medications suggests a comparable baseline between the groups, we were unable to fully standardize patient characteristics due to these missing records. Therefore, although both drugs are prescribed for similar indications, we cannot fully exclude the possibility that the observed safety signals may be influenced by prior treatments or other unmeasured variables. Nevertheless, the strength of this study lies in its large sample size and real-world environment, capturing rare or delayed AE signals that may be difficult to detect in tightly controlled clinical trials, offering valuable perspectives on the safety comparison of the two drugs.

In conclusion, our study demonstrates that although guselkumab and risankizumab both belong to the IL-23 inhibitor class, their safety signal profiles differ significantly in the real world. These differences may stem from factors such as distinct indication populations, report sources, and potential drug-specific effects. The multiple signals of concern identified in this study, particularly risankizumab’s cancer signals and guselkumab’s efficacy-related and emerging autoimmune signals, provide specific directions for clinical risk management and underscore the need for continued post-market pharmacovigilance. Future research should combine multi-source data, such as electronic health records, to further validate and conduct causal inference on these signals.

## 5 Conclusion

Our study provides real-world confirmation of known AEs associated with IL-23 inhibitors, while also identifying distinct safety profiles that necessitate drug-specific risk management. Specifically, risankizumab exhibited stronger signals for severe organic pathologies, including skin cancer and gastrointestinal obstruction. Consequently, clinicians should exercise heightened vigilance when prescribing risankizumab, particularly for elderly patients (≥65 years), who demonstrated significantly elevated risks for these specific AEs.

In contrast, guselkumab presented a relatively more favorable safety profile regarding organic lesions but was predominantly associated with medication management challenges, such as dose omission and accidental exposure. Therefore, the clinical focus for guselkumab should prioritize patient education on proper administration to ensure adherence and avoid potential therapeutic compromises.

These findings underscore the importance of individualized therapy. Factors such as patient age, comorbidities, and the specific safety signals identified in this study should guide treatment selection. As real-world evidence continues to accumulate, these insights will aid in optimizing treatment strategies, maximizing patient benefits, and minimizing drug-specific AEs.

## Data Availability

The original contributions presented in the study are included in the article/Supplementary Material, further inquiries can be directed to the corresponding author.
