# Supplementary material for: Comparative safety profiles of risankizumab versus guselkumab: a pharmacovigilance study based on the FAERS database
Source: Front Pharmacol. 2026 Feb 18;17:1765114. doi: 10.3389/fphar.2026.1765114 (PMC12957254; doi:10.3389/fphar.2026.1765114)
Supplement: Supplementary file 1 [file Table1.docx]

Supplementary Data

**Table S1.** Ratio imbalance measurement algorithm.

| Item | Reports with the target AEs | All other AEs | Total |
| --- | --- | --- | --- |
| Reports with the target drug | a | b | a+b |
| All other drugs | c | d | c+d |
| Total | a+c | b+d | a+b+c+d |

**Table S2.** Principle of dis-proportionality measure and standard of signal detection.

| Algorithms | Calculation formula | Criteria |
| --- | --- | --- |
| ROR | $ROR=\frac{a/c}{b/d}=\frac{ad}{bc}$ $95\%CI=e^{\ln(ROR)\pm1.96\sqrt{\frac{1}{a}+\frac{1}{b}+\frac{1}{c}+\frac{1}{d}}}$   | (1) a ≥ 3  (2) ROR ≥ 2  (3) 95%CI > 1 |
| PRR | $PRR=\frac{a/\left(a+b\right)}{c/\left(c+d\right)}=\frac{a\left(c+d\right)}{c\left(a+b\right)}$ $\chi^{2}=\frac{\left(\left\vert ad-bc\right\vert-\frac{n}{2}\right)^{2}n}{\left(a+b\right)\left(a+c\right)\left(c+d\right)\left(b+d\right)}$    n= a + b + c + d | (1)$a\geq3$  (2) PRR ≥ 2  (3)$\chi^{2}\geq4$ |
| BCPNN | $E(IC)=\log_{2}\frac{(C_{xy}+\gamma_{11})(C+\alpha)(C+\beta)}{(C+\gamma)(C_{x}+\alpha_{1})(C_{y}+\beta_{1})}$ | (1) a ≥ 3  (2) IC-2SD > 0 |
|  | $V(IC)=\frac{1}{(\ln2)^{2}}\left\{\left(\frac{C-C_{xy}+\gamma-\gamma_{11}}{(C_{xy}+\gamma_{11})(1+C+\gamma)}\right)+\left(\frac{C-C_{x}+\alpha-\alpha_{1}}{(C_{x}+\alpha_{1})(1+C+\alpha)}\right)+\left(\frac{C-C_{y}+\beta-\beta_{1}}{(C_{y}+\beta_{1})(1+C+\beta)}\right)\right\}$ $\gamma=\gamma_{11}\frac{(C+\alpha)(C+\beta)}{(C_{x}+\alpha_{1})(C_{y}+\beta_{1})}$ $IC-2SD=E(IC)-2\sqrt{V(IC)}$ $\alpha_{1}=\beta_{1}=1;\alpha=\beta=2;\gamma_{11}=1;$ $C=a+b+c+d;C_{x}=a+b;C_{y}=a+c;C_{xy}=a$         | |

Abbreviations: ROR: Reporting odds ratio; PRR：Proportional Reporting Ratio ; BCPNN: Bayesian confidence propagation neural network; CI: Confidence Interval; IC: Information Component

**Table S3** Signal strength of adverse events of risankizumab ranked by report frequency at the preferred terms level

| **SOC** | **Preferred Terms** | **N** | **ROR(95% CI)** | **PRR(95% CI)** | **chisq** | **IC(IC025)** |
| --- | --- | --- | --- | --- | --- | --- |
| skin and subcutaneous tissue disorders | pruritus | 2080 | 2.8(2.68, 2.92) | 2.76(2.65, 2.87) | 2326.71 | 1.45(1.39) |
| infections and infestations | covid-19 | 1892 | 2.67(2.55, 2.79) | 2.64(2.54, 2.75) | 1916.86 | 1.39(1.32) |
| musculoskeletal and connective tissue disorders | arthralgia | 1883 | 2.38(2.28, 2.49) | 2.36(2.27, 2.45) | 1470.57 | 1.23(1.16) |
| injury, poisoning and procedural complications | fall | 1364 | 2.35(2.22, 2.48) | 2.33(2.2, 2.47) | 1031.55 | 1.21(1.14) |
| infections and infestations | nasopharyngitis | 997 | 2.87(2.69, 3.05) | 2.85(2.69, 3.02) | 1187.19 | 1.5(1.41) |
| skin and subcutaneous tissue disorders | erythema | 882 | 2.09(1.95, 2.23) | 2.08(1.96, 2.21) | 489.69 | 1.05(0.95) |
| nervous system disorders | cerebrovascular accident | 823 | 3.67(3.42, 3.93) | 3.65(3.37, 3.95) | 1561.16 | 1.85(1.75) |
| skin and subcutaneous tissue disorders | skin disorder | 751 | 12.41(11.53, 13.36) | 12.33(11.4, 13.34) | 7421.76 | 3.55(3.45) |
| general disorders and administration site conditions | illness | 736 | 2.08(1.94, 2.24) | 2.07(1.91, 2.24) | 407.08 | 1.05(0.94) |
| infections and infestations | urinary tract infection | 724 | 2.21(2.05, 2.38) | 2.2(2.03, 2.38) | 470.63 | 1.13(1.02) |
| neoplasms benign, malignant and unspecified (incl cysts and polyps) | skin cancer | 692 | 12.62(11.69, 13.63) | 12.55(11.6, 13.57) | 6975.21 | 3.58(3.47) |
| general disorders and administration site conditions | unevaluable event | 659 | 5.4(5, 5.84) | 5.38(4.97, 5.82) | 2295.42 | 2.4(2.29) |
| gastrointestinal disorders | intestinal obstruction | 652 | 10.33(9.55, 11.18) | 10.28(9.5, 11.12) | 5225.33 | 3.3(3.19) |
| cardiac disorders | myocardial infarction | 630 | 3.81(3.52, 4.12) | 3.79(3.5, 4.1) | 1275.11 | 1.9(1.79) |
| infections and infestations | influenza | 615 | 2.82(2.61, 3.06) | 2.81(2.6, 3.04) | 711.1 | 1.48(1.37) |
| eye disorders | cataract | 604 | 5.29(4.88, 5.73) | 5.26(4.86, 5.69) | 2041.1 | 2.37(2.25) |
| general disorders and administration site conditions | injection site haemorrhage | 585 | 4.03(3.71, 4.37) | 4.01(3.71, 4.34) | 1301.69 | 1.99(1.87) |
| neoplasms benign, malignant and unspecified (incl cysts and polyps) | neoplasm malignant | 575 | 3.64(3.35, 3.95) | 3.62(3.35, 3.92) | 1075.64 | 1.84(1.72) |
| nervous system disorders | loss of consciousness | 494 | 2.5(2.29, 2.73) | 2.5(2.27, 2.76) | 438.67 | 1.31(1.18) |
| skin and subcutaneous tissue disorders | skin exfoliation | 484 | 2.51(2.29, 2.74) | 2.5(2.27, 2.76) | 432.38 | 1.31(1.18) |
| renal and urinary disorders | nephrolithiasis | 460 | 5.17(4.71, 5.67) | 5.15(4.67, 5.68) | 1505.55 | 2.34(2.21) |
| general disorders and administration site conditions | therapeutic response shortened | 436 | 4.79(4.36, 5.27) | 4.78(4.33, 5.27) | 1277.2 | 2.23(2.1) |
| musculoskeletal and connective tissue disorders | arthritis | 435 | 3.15(2.87, 3.47) | 3.14(2.85, 3.46) | 628 | 1.64(1.5) |
| infections and infestations | sinusitis | 418 | 2.26(2.05, 2.49) | 2.26(2.05, 2.49) | 289.94 | 1.17(1.03) |
| investigations | sars-cov-2 test positive | 301 | 5.57(4.96, 6.24) | 5.55(4.93, 6.24) | 1097.63 | 2.44(2.28) |
| general disorders and administration site conditions | hernia | 295 | 8.26(7.35, 9.28) | 8.24(7.33, 9.27) | 1812.07 | 3(2.83) |
| infections and infestations | upper respiratory tract infection | 293 | 3.78(3.37, 4.24) | 3.77(3.35, 4.24) | 588.05 | 1.9(1.73) |
| neoplasms benign, malignant and unspecified (incl cysts and polyps) | basal cell carcinoma | 285 | 9.9(8.79, 11.14) | 9.87(8.77, 11.1) | 2178.68 | 3.25(3.08) |
| psychiatric disorders | stress | 275 | 2.14(1.9, 2.41) | 2.14(1.9, 2.41) | 165.68 | 1.09(0.92) |
| musculoskeletal and connective tissue disorders | osteoarthritis | 267 | 3.88(3.44, 4.38) | 3.87(3.44, 4.35) | 559.88 | 1.94(1.76) |
| metabolism and nutrition disorders | diabetes mellitus | 265 | 2.28(2.02, 2.58) | 2.28(2.03, 2.56) | 188.6 | 1.18(1.01) |
| general disorders and administration site conditions | inflammation | 264 | 2.56(2.27, 2.89) | 2.56(2.28, 2.88) | 247.4 | 1.34(1.17) |
| injury, poisoning and procedural complications | procedural pain | 259 | 5.56(4.92, 6.29) | 5.55(4.93, 6.24) | 944.04 | 2.44(2.27) |
| nervous system disorders | burning sensation | 257 | 2.34(2.07, 2.65) | 2.34(2.08, 2.63) | 195.59 | 1.22(1.04) |
| general disorders and administration site conditions | injection site bruising | 246 | 2.05(1.81, 2.32) | 2.05(1.82, 2.31) | 130.51 | 1.03(0.85) |
| infections and infestations | bronchitis | 240 | 2.05(1.81, 2.33) | 2.05(1.82, 2.31) | 128.04 | 1.03(0.85) |
| neoplasms benign, malignant and unspecified (incl cysts and polyps) | breast cancer female | 239 | 5.13(4.51, 5.83) | 5.12(4.46, 5.87) | 774.95 | 2.33(2.15) |
| skin and subcutaneous tissue disorders | skin discolouration | 239 | 2.91(2.56, 3.31) | 2.91(2.54, 3.34) | 296.01 | 1.53(1.35) |
| skin and subcutaneous tissue disorders | skin plaque | 233 | 16.29(14.26, 18.6) | 16.25(14.17, 18.64) | 3112.78 | 3.93(3.74) |
| infections and infestations | cellulitis | 210 | 2.51(2.19, 2.88) | 2.51(2.19, 2.88) | 189.07 | 1.32(1.12) |
| respiratory, thoracic and mediastinal disorders | chronic obstructive pulmonary disease | 209 | 2.5(2.18, 2.86) | 2.49(2.17, 2.86) | 185.3 | 1.31(1.11) |
| infections and infestations | diverticulitis | 201 | 4.06(3.53, 4.66) | 4.05(3.53, 4.65) | 453.98 | 2(1.8) |
| hepatobiliary disorders | hepatic cirrhosis | 192 | 6.47(5.6, 7.47) | 6.46(5.63, 7.41) | 861.62 | 2.66(2.45) |
| injury, poisoning and procedural complications | limb injury | 192 | 3.31(2.87, 3.82) | 3.31(2.89, 3.8) | 305.21 | 1.71(1.51) |
| gastrointestinal disorders | small intestinal obstruction | 188 | 10.31(8.9, 11.93) | 10.29(8.97, 11.8) | 1508.77 | 3.31(3.1) |
| neoplasms benign, malignant and unspecified (incl cysts and polyps) | malignant melanoma | 187 | 7.08(6.12, 8.19) | 7.07(6.16, 8.11) | 945.56 | 2.78(2.57) |
| injury, poisoning and procedural complications | hip fracture | 185 | 3.25(2.81, 3.76) | 3.25(2.83, 3.73) | 284.17 | 1.69(1.48) |
| injury, poisoning and procedural complications | post procedural complication | 184 | 7.05(6.09, 8.17) | 7.04(6.14, 8.08) | 925.47 | 2.78(2.57) |
| infections and infestations | localised infection | 180 | 3.88(3.35, 4.5) | 3.88(3.32, 4.54) | 378.21 | 1.94(1.73) |
| musculoskeletal and connective tissue disorders | rotator cuff syndrome | 177 | 9.24(7.95, 10.74) | 9.22(7.88, 10.79) | 1247.18 | 3.15(2.94) |
| immune system disorders | immunodeficiency | 171 | 5.13(4.41, 5.97) | 5.13(4.39, 6) | 555.35 | 2.33(2.11) |
| hepatobiliary disorders | cholelithiasis | 170 | 4.1(3.53, 4.78) | 4.1(3.5, 4.8) | 391.41 | 2.02(1.8) |
| pregnancy, puerperium and perinatal conditions | abortion spontaneous | 166 | 2.6(2.23, 3.03) | 2.6(2.22, 3.04) | 161.47 | 1.37(1.15) |
| musculoskeletal and connective tissue disorders | musculoskeletal disorder | 165 | 5.18(4.44, 6.04) | 5.17(4.42, 6.05) | 542.67 | 2.34(2.12) |
| cardiac disorders | cardiac failure congestive | 165 | 2.38(2.04, 2.77) | 2.37(2.03, 2.77) | 130.01 | 1.24(1.02) |
| general disorders and administration site conditions | injection site papule | 164 | 26.73(22.73, 31.42) | 26.69(22.82, 31.22) | 3627.78 | 4.58(4.35) |
| skin and subcutaneous tissue disorders | skin fissures | 164 | 3.63(3.11, 4.24) | 3.63(3.1, 4.25) | 307.54 | 1.84(1.62) |
| infections and infestations | tuberculosis | 163 | 7.26(6.21, 8.49) | 7.25(6.2, 8.48) | 851.34 | 2.82(2.59) |
| gastrointestinal disorders | frequent bowel movements | 161 | 3.52(3.01, 4.11) | 3.51(3, 4.11) | 285.38 | 1.8(1.57) |
| respiratory, thoracic and mediastinal disorders | respiratory disorder | 161 | 3.12(2.67, 3.64) | 3.11(2.66, 3.64) | 227.75 | 1.62(1.4) |
| nervous system disorders | carpal tunnel syndrome | 159 | 6.48(5.53, 7.58) | 6.47(5.53, 7.57) | 714.83 | 2.66(2.43) |
| general disorders and administration site conditions | impaired healing | 159 | 3.27(2.8, 3.83) | 3.27(2.8, 3.83) | 247.23 | 1.7(1.47) |
| general disorders and administration site conditions | cyst | 158 | 7.27(6.2, 8.52) | 7.26(6.21, 8.49) | 826.48 | 2.82(2.59) |
| gastrointestinal disorders | pancreatitis | 154 | 2.19(1.87, 2.56) | 2.19(1.87, 2.56) | 98.24 | 1.12(0.89) |
| musculoskeletal and connective tissue disorders | intervertebral disc protrusion | 151 | 4.59(3.9, 5.39) | 4.58(3.92, 5.36) | 414.58 | 2.17(1.94) |
| infections and infestations | ear infection | 150 | 2.86(2.43, 3.36) | 2.86(2.44, 3.35) | 178.72 | 1.5(1.27) |
| skin and subcutaneous tissue disorders | rash macular | 147 | 2.07(1.76, 2.43) | 2.07(1.77, 2.42) | 80.17 | 1.04(0.81) |
| injury, poisoning and procedural complications | head injury | 145 | 2.68(2.28, 3.16) | 2.68(2.29, 3.13) | 150.77 | 1.41(1.18) |
| infections and infestations | abscess | 141 | 5.16(4.37, 6.1) | 5.16(4.33, 6.16) | 462.34 | 2.34(2.1) |
| injury, poisoning and procedural complications | scar | 141 | 5(4.23, 5.9) | 4.99(4.18, 5.95) | 440.43 | 2.29(2.05) |
| general disorders and administration site conditions | obstruction | 139 | 15.32(12.9, 18.2) | 15.31(12.83, 18.26) | 1741.24 | 3.85(3.6) |
| cardiac disorders | coronary artery occlusion | 139 | 10.18(8.59, 12.06) | 10.17(8.53, 12.13) | 1099.64 | 3.29(3.04) |
| musculoskeletal and connective tissue disorders | fistula | 138 | 7.37(6.22, 8.74) | 7.37(6.18, 8.79) | 735.41 | 2.84(2.6) |
| skin and subcutaneous tissue disorders | rash papular | 137 | 3.4(2.87, 4.03) | 3.4(2.85, 4.06) | 228.63 | 1.75(1.51) |
| infections and infestations | staphylococcal infection | 136 | 2.88(2.43, 3.41) | 2.87(2.41, 3.42) | 164.15 | 1.51(1.27) |
| infections and infestations | post procedural infection | 130 | 8.2(6.88, 9.77) | 8.19(6.87, 9.77) | 792.21 | 2.99(2.74) |
| infections and infestations | kidney infection | 129 | 3.29(2.76, 3.91) | 3.28(2.75, 3.91) | 201.98 | 1.7(1.45) |
| injury, poisoning and procedural complications | ankle fracture | 126 | 4.62(3.88, 5.52) | 4.62(3.87, 5.51) | 350.23 | 2.18(1.93) |
| injury, poisoning and procedural complications | lower limb fracture | 125 | 3.83(3.21, 4.58) | 3.83(3.21, 4.57) | 257.29 | 1.92(1.67) |
| injury, poisoning and procedural complications | upper limb fracture | 122 | 3.6(3.01, 4.31) | 3.6(3.02, 4.29) | 225.29 | 1.83(1.57) |
| ear and labyrinth disorders | deafness | 121 | 2.38(1.99, 2.85) | 2.38(2, 2.84) | 96.05 | 1.24(0.99) |
| injury, poisoning and procedural complications | skin laceration | 120 | 4.07(3.39, 4.87) | 4.06(3.4, 4.84) | 272.17 | 2(1.74) |
| neoplasms benign, malignant and unspecified (incl cysts and polyps) | colon cancer | 119 | 3.94(3.29, 4.72) | 3.94(3.3, 4.7) | 256.27 | 1.96(1.7) |
| nervous system disorders | transient ischaemic attack | 115 | 2.69(2.23, 3.23) | 2.68(2.25, 3.2) | 120.07 | 1.41(1.15) |
| injury, poisoning and procedural complications | accident | 110 | 4.73(3.92, 5.71) | 4.73(3.89, 5.75) | 316.72 | 2.22(1.95) |
| infections and infestations | clostridium difficile infection | 108 | 2.48(2.05, 2.99) | 2.47(2.03, 3) | 93.83 | 1.3(1.02) |
| injury, poisoning and procedural complications | meniscus injury | 107 | 9.28(7.65, 11.26) | 9.28(7.63, 11.29) | 759.05 | 3.16(2.88) |
| gastrointestinal disorders | gastrointestinal inflammation | 107 | 7.79(6.42, 9.45) | 7.78(6.4, 9.46) | 611.64 | 2.92(2.64) |
| injury, poisoning and procedural complications | rib fracture | 107 | 2.88(2.38, 3.48) | 2.88(2.37, 3.5) | 129.26 | 1.51(1.24) |
| skin and subcutaneous tissue disorders | skin ulcer | 107 | 2.38(1.97, 2.88) | 2.38(1.96, 2.9) | 84.44 | 1.24(0.97) |
| musculoskeletal and connective tissue disorders | back disorder | 102 | 3.68(3.03, 4.48) | 3.68(3.03, 4.48) | 196.03 | 1.86(1.58) |
| neoplasms benign, malignant and unspecified (incl cysts and polyps) | squamous cell carcinoma | 101 | 6.12(5.02, 7.46) | 6.12(5.03, 7.45) | 421.05 | 2.58(2.3) |
| infections and infestations | appendicitis | 101 | 6.01(4.93, 7.33) | 6.01(4.94, 7.31) | 410.79 | 2.56(2.27) |
| injury, poisoning and procedural complications | foot fracture | 101 | 3.01(2.48, 3.67) | 3.01(2.47, 3.66) | 133.96 | 1.58(1.3) |
| respiratory, thoracic and mediastinal disorders | pulmonary thrombosis | 100 | 4.27(3.5, 5.2) | 4.26(3.5, 5.18) | 245.17 | 2.07(1.79) |
| injury, poisoning and procedural complications | joint injury | 99 | 2.55(2.09, 3.1) | 2.54(2.09, 3.09) | 91.75 | 1.34(1.05) |
| neoplasms benign, malignant and unspecified (incl cysts and polyps) | squamous cell carcinoma of skin | 97 | 7.78(6.35, 9.52) | 7.77(6.39, 9.45) | 553.23 | 2.92(2.63) |
| respiratory, thoracic and mediastinal disorders | sinus disorder | 96 | 2.65(2.16, 3.24) | 2.64(2.17, 3.21) | 97.02 | 1.39(1.1) |
| respiratory, thoracic and mediastinal disorders | respiratory tract congestion | 94 | 3.63(2.96, 4.45) | 3.62(2.98, 4.4) | 175.75 | 1.84(1.55) |
| neoplasms benign, malignant and unspecified (incl cysts and polyps) | lymphoma | 94 | 3.11(2.54, 3.82) | 3.11(2.56, 3.78) | 133.03 | 1.62(1.33) |
| injury, poisoning and procedural complications | spinal fracture | 92 | 2.41(1.96, 2.95) | 2.4(1.97, 2.92) | 74.71 | 1.26(0.96) |
| general disorders and administration site conditions | ulcer | 90 | 3.41(2.77, 4.2) | 3.41(2.75, 4.23) | 150.94 | 1.75(1.46) |
| infections and infestations | pharyngitis streptococcal | 86 | 4.37(3.53, 5.42) | 4.37(3.52, 5.42) | 219.45 | 2.11(1.8) |
| infections and infestations | bacterial infection | 86 | 2.42(1.96, 2.99) | 2.42(1.95, 3) | 70.75 | 1.26(0.96) |
| vascular disorders | arterial occlusive disease | 84 | 8.85(7.12, 11.01) | 8.85(7.13, 10.98) | 562.64 | 3.1(2.78) |
| musculoskeletal and connective tissue disorders | joint range of motion decreased | 84 | 4.08(3.29, 5.06) | 4.08(3.29, 5.06) | 191.8 | 2.01(1.7) |
| infections and infestations | postoperative wound infection | 82 | 6.22(5, 7.75) | 6.22(5.01, 7.72) | 349.55 | 2.6(2.29) |
| musculoskeletal and connective tissue disorders | exostosis | 80 | 7.17(5.74, 8.96) | 7.17(5.78, 8.9) | 411.65 | 2.8(2.48) |
| respiratory, thoracic and mediastinal disorders | sinus congestion | 80 | 3.8(3.05, 4.74) | 3.8(3.06, 4.71) | 162.18 | 1.91(1.59) |
| investigations | blood iron decreased | 80 | 3.16(2.53, 3.94) | 3.16(2.55, 3.92) | 116.31 | 1.64(1.33) |
| neoplasms benign, malignant and unspecified (incl cysts and polyps) | melanocytic naevus | 79 | 9.74(7.77, 12.2) | 9.73(7.84, 12.07) | 593.41 | 3.23(2.91) |
| nervous system disorders | nerve compression | 79 | 4.77(3.81, 5.96) | 4.76(3.84, 5.91) | 230.16 | 2.23(1.91) |
| gastrointestinal disorders | intestinal stenosis | 78 | 16.46(13.08, 20.72) | 16.45(13, 20.81) | 1055.34 | 3.95(3.62) |
| gastrointestinal disorders | intestinal perforation | 78 | 4.38(3.5, 5.48) | 4.38(3.53, 5.43) | 199.3 | 2.11(1.79) |
| hepatobiliary disorders | hepatic steatosis | 78 | 2.59(2.08, 3.24) | 2.59(2.09, 3.21) | 75.49 | 1.36(1.04) |
| investigations | mycobacterium tuberculosis complex test positive | 77 | 17.45(13.84, 22.01) | 17.44(13.78, 22.06) | 1108.18 | 4.02(3.69) |
| injury, poisoning and procedural complications | tendon rupture | 77 | 4.71(3.76, 5.9) | 4.71(3.72, 5.96) | 220.3 | 2.21(1.89) |
| skin and subcutaneous tissue disorders | scab | 77 | 3.73(2.98, 4.67) | 3.73(3.01, 4.63) | 151.12 | 1.88(1.56) |
| general disorders and administration site conditions | injection site discharge | 76 | 6.6(5.25, 8.29) | 6.6(5.22, 8.35) | 350.65 | 2.69(2.36) |
| injury, poisoning and procedural complications | wrist fracture | 75 | 3.59(2.85, 4.51) | 3.58(2.83, 4.53) | 137.59 | 1.83(1.5) |
| gastrointestinal disorders | gastrointestinal sounds abnormal | 74 | 8.94(7.08, 11.27) | 8.93(7.06, 11.3) | 501.41 | 3.11(2.78) |
| nervous system disorders | sciatica | 73 | 2.79(2.22, 3.52) | 2.79(2.21, 3.53) | 82.98 | 1.47(1.14) |

Asterisks (*) indicate novel signals not previously documented in the drug label or clinical trials. N, case number; ROR, reports odds ratio; IC, information component; IC025, the lower limit of the 95% CI of IC; CI, confidence interval.

**Table S4** Signal strength of adverse events of guselkumab ranked by report frequency at the preferred terms level

| **SOC** | **Preferred Terms** | **N** | **ROR(95% CI)** | **PRR(95% CI)** | **chisq** | **IC(IC025)** |
| --- | --- | --- | --- | --- | --- | --- |
| injury, poisoning and procedural complications | product dose omission issue | 8242 | 41.23(40.22, 42.27) | 32.36(31.73, 33) | 242939.36 | 4.96(4.93) |
| injury, poisoning and procedural complications | accidental exposure to product | 2569 | 32.33(31.04, 33.68) | 30.18(29.02, 31.39) | 70137.22 | 4.87(4.81) |
| injury, poisoning and procedural complications | product storage error | 912 | 10.71(10.02, 11.44) | 10.47(9.87, 11.1) | 7736.23 | 3.37(3.28) |
| injury, poisoning and procedural complications | inappropriate schedule of product administration | 714 | 4.2(3.9, 4.53) | 4.14(3.83, 4.48) | 1702.02 | 2.05(1.94) |
| infections and infestations | pneumonia | 682 | 3.36(3.11, 3.62) | 3.32(3.07, 3.59) | 1104.73 | 1.73(1.62) |
| musculoskeletal and connective tissue disorders | arthralgia | 577 | 2.22(2.04, 2.41) | 2.2(2.03, 2.38) | 378.21 | 1.13(1.02) |
| injury, poisoning and procedural complications | underdose | 560 | 11.03(10.14, 11.99) | 10.88(10.06, 11.77) | 4966.43 | 3.43(3.31) |
| general disorders and administration site conditions | condition aggravated | 479 | 2.19(2, 2.4) | 2.17(1.97, 2.39) | 304.57 | 1.12(0.99) |
| infections and infestations | covid-19 | 406 | 2.1(1.91, 2.32) | 2.09(1.89, 2.31) | 231.74 | 1.06(0.92) |
| general disorders and administration site conditions | injection site haemorrhage | 264 | 5.52(4.89, 6.23) | 5.49(4.88, 6.18) | 964.09 | 2.45(2.27) |
| infections and infestations | lower respiratory tract infection | 226 | 7.48(6.56, 8.53) | 7.44(6.49, 8.53) | 1249.17 | 2.88(2.69) |
| general disorders and administration site conditions | therapeutic product effect decreased | 169 | 4.09(3.52, 4.76) | 4.08(3.49, 4.77) | 391.13 | 2.02(1.8) |
| general disorders and administration site conditions | injection site reaction | 167 | 4.88(4.19, 5.68) | 4.86(4.15, 5.69) | 509.9 | 2.28(2.06) |
| injury, poisoning and procedural complications | exposure during pregnancy | 109 | 2.54(2.11, 3.07) | 2.54(2.09, 3.09) | 101.4 | 1.34(1.07) |
| injury, poisoning and procedural complications | product administration error | 106 | 5.22(4.31, 6.32) | 5.21(4.28, 6.34) | 358.36 | 2.37(2.1) |
| general disorders and administration site conditions | therapeutic response decreased | 104 | 3.03(2.5, 3.68) | 3.03(2.49, 3.69) | 140.71 | 1.59(1.32) |
| infections and infestations | cellulitis | 97 | 3.27(2.68, 4) | 3.27(2.69, 3.98) | 152.11 | 1.7(1.42) |
| investigations | hepatic enzyme increased | 96 | 2.33(1.9, 2.84) | 2.32(1.91, 2.82) | 72.14 | 1.21(0.93) |
| general disorders and administration site conditions | injection site swelling | 94 | 2.32(1.89, 2.84) | 2.31(1.9, 2.81) | 69.96 | 1.21(0.92) |
| musculoskeletal and connective tissue disorders | arthritis | 92 | 2.01(1.64, 2.47) | 2.01(1.65, 2.45) | 46.79 | 1.01(0.71) |
| infections and infestations | bronchitis | 90 | 2.17(1.76, 2.67) | 2.17(1.75, 2.69) | 56.44 | 1.11(0.82) |
| injury, poisoning and procedural complications | incorrect route of product administration | 83 | 5.01(4.04, 6.22) | 5.01(4.04, 6.22) | 264.57 | 2.32(2.01) |
| infections and infestations | upper respiratory tract infection | 78 | 2.85(2.28, 3.56) | 2.85(2.3, 3.54) | 93.41 | 1.51(1.19) |
| general disorders and administration site conditions | therapy non-responder | 76 | 2.04(1.63, 2.56) | 2.04(1.64, 2.53) | 40.39 | 1.03(0.71) |
| skin and subcutaneous tissue disorders | skin disorder | 66 | 3.14(2.46, 4) | 3.13(2.47, 3.96) | 95.57 | 1.64(1.3) |
| general disorders and administration site conditions | complication associated with device | 59 | 4.98(3.85, 6.43) | 4.97(3.85, 6.41) | 186.18 | 2.31(1.94) |
| infections and infestations | respiratory tract infection | 57 | 3.26(2.51, 4.23) | 3.26(2.53, 4.21) | 88.94 | 1.7(1.33) |
| injury, poisoning and procedural complications | poor quality product administered | 55 | 5.47(4.19, 7.13) | 5.46(4.15, 7.18) | 199.13 | 2.44(2.06) |
| infections and infestations | tuberculosis | 52 | 6.69(5.09, 8.79) | 6.68(5.08, 8.79) | 249.14 | 2.73(2.34) |
| injury, poisoning and procedural complications | product dispensing error | 49 | 2.17(1.64, 2.87) | 2.17(1.65, 2.86) | 30.76 | 1.11(0.71) |
| infections and infestations | kidney infection | 44 | 3.37(2.5, 4.53) | 3.36(2.5, 4.51) | 72.84 | 1.75(1.32) |
| general disorders and administration site conditions | injection site rash | 43 | 2.57(1.91, 3.47) | 2.57(1.92, 3.45) | 41.15 | 1.36(0.93) |
| infections and infestations | fungal infection | 43 | 2.1(1.56, 2.83) | 2.1(1.57, 2.82) | 24.67 | 1.07(0.64) |
| injury, poisoning and procedural complications | device use issue | 41 | 2.4(1.76, 3.26) | 2.4(1.75, 3.28) | 33.28 | 1.26(0.82) |
| skin and subcutaneous tissue disorders | skin plaque | 39 | 8.16(5.95, 11.18) | 8.15(5.96, 11.15) | 242.35 | 3.01(2.57) |
| investigations | sars-cov-2 test positive | 39 | 2.63(1.92, 3.6) | 2.62(1.91, 3.59) | 39.13 | 1.39(0.94) |
| investigations | mycobacterium tuberculosis complex test positive | 35 | 20.94(14.97, 29.28) | 20.92(14.99, 29.19) | 647.78 | 4.35(3.88) |
| infections and infestations | oral herpes | 34 | 2.55(1.82, 3.57) | 2.55(1.83, 3.56) | 31.86 | 1.35(0.87) |
| hepatobiliary disorders | hepatic cirrhosis | 31 | 2.96(2.08, 4.21) | 2.96(2.08, 4.21) | 40.02 | 1.56(1.06) |
| musculoskeletal and connective tissue disorders | joint stiffness | 31 | 2.1(1.48, 2.99) | 2.1(1.48, 2.99) | 17.9 | 1.07(0.57) |
| investigations | liver function test abnormal | 27 | 2.74(1.88, 4) | 2.74(1.89, 3.98) | 29.69 | 1.45(0.91) |
| immune system disorders | decreased immune responsiveness | 25 | 3.25(2.2, 4.82) | 3.25(2.2, 4.81) | 38.81 | 1.7(1.14) |
| neoplasms benign, malignant and unspecified (incl cysts and polyps) | malignant melanoma | 25 | 2.71(1.83, 4.02) | 2.71(1.83, 4.01) | 26.94 | 1.44(0.88) |
| neoplasms benign, malignant and unspecified (incl cysts and polyps) | colon cancer | 23 | 2.33(1.55, 3.51) | 2.33(1.54, 3.52) | 17.47 | 1.22(0.64) |
| infections and infestations | latent tuberculosis | 22 | 14.92(9.79, 22.75) | 14.91(9.69, 22.95) | 280.6 | 3.87(3.28) |
| skin and subcutaneous tissue disorders | pemphigoid | 19 | 3.47(2.21, 5.44) | 3.46(2.2, 5.43) | 33.17 | 1.79(1.15) |
| immune system disorders | immune system disorder | 19 | 2.19(1.4, 3.44) | 2.19(1.4, 3.44) | 12.25 | 1.13(0.5) |
| infections and infestations | herpes virus infection | 17 | 5.05(3.13, 8.14) | 5.05(3.15, 8.08) | 54.86 | 2.33(1.66) |
| infections and infestations | hepatitis c | 17 | 2.75(1.71, 4.43) | 2.75(1.72, 4.4) | 18.84 | 1.46(0.79) |
| skin and subcutaneous tissue disorders | dermatitis exfoliative generalised | 16 | 4.86(2.97, 7.94) | 4.85(2.97, 7.92) | 48.68 | 2.27(1.58) |
| general disorders and administration site conditions | injection site nodule | 16 | 3.14(1.92, 5.12) | 3.13(1.92, 5.11) | 23.18 | 1.64(0.96) |
| neoplasms benign, malignant and unspecified (incl cysts and polyps) | squamous cell carcinoma | 16 | 2.75(1.68, 4.49) | 2.75(1.68, 4.49) | 17.7 | 1.45(0.77) |
| infections and infestations | pneumonia bacterial | 16 | 2.36(1.44, 3.85) | 2.36(1.45, 3.85) | 12.44 | 1.23(0.55) |
| infections and infestations | herpes simplex | 15 | 4.63(2.79, 7.69) | 4.63(2.78, 7.71) | 42.46 | 2.2(1.5) |
| infections and infestations | appendicitis | 14 | 2.45(1.45, 4.14) | 2.45(1.44, 4.16) | 11.97 | 1.29(0.56) |
| infections and infestations | pharyngitis streptococcal | 14 | 2.12(1.25, 3.58) | 2.12(1.25, 3.6) | 8.23 | 1.08(0.35) |
| injury, poisoning and procedural complications | procedural complication | 13 | 4.98(2.89, 8.6) | 4.98(2.88, 8.62) | 41.14 | 2.31(1.55) |
| injury, poisoning and procedural complications | injection related reaction | 12 | 8.9(5.04, 15.72) | 8.9(5.04, 15.71) | 83.26 | 3.14(2.35) |
| infections and infestations | tonsillitis | 12 | 3.66(2.07, 6.45) | 3.66(2.07, 6.46) | 23.07 | 1.87(1.08) |
| skin and subcutaneous tissue disorders | vitiligo | 11 | 6.54(3.62, 11.84) | 6.54(3.63, 11.77) | 51.25 | 2.7(1.88) |
| infections and infestations | erysipelas | 11 | 3.42(1.89, 6.18) | 3.42(1.9, 6.16) | 18.72 | 1.77(0.95) |
| general disorders and administration site conditions | injection site vesicles | 11 | 3.05(1.69, 5.52) | 3.05(1.69, 5.49) | 15.12 | 1.61(0.79) |
| infections and infestations | suspected covid-19 | 11 | 3.03(1.68, 5.49) | 3.03(1.68, 5.46) | 14.94 | 1.6(0.78) |
| skin and subcutaneous tissue disorders | erythrodermic psoriasis | 10 | 30.58(16.27, 57.48) | 30.58(16.33, 57.26) | 276.09 | 4.88(4.02) |
| infections and infestations | fungal skin infection | 10 | 3.8(2.04, 7.07) | 3.8(2.03, 7.11) | 20.51 | 1.92(1.06) |
| general disorders and administration site conditions | injection site discharge | 10 | 3.03(1.63, 5.64) | 3.03(1.62, 5.67) | 13.56 | 1.6(0.74) |
| metabolism and nutrition disorders | overweight | 10 | 2.79(1.5, 5.19) | 2.79(1.49, 5.22) | 11.43 | 1.48(0.62) |
| injury, poisoning and procedural complications | wrong schedule | 9 | 8.72(4.52, 16.82) | 8.72(4.48, 16.98) | 60.88 | 3.11(2.21) |
| neoplasms benign, malignant and unspecified (incl cysts and polyps) | endometrial cancer | 9 | 7.24(3.76, 13.96) | 7.24(3.79, 13.82) | 48 | 2.85(1.95) |
| neoplasms benign, malignant and unspecified (incl cysts and polyps) | hodgkin's disease | 9 | 3.7(1.92, 7.13) | 3.7(1.94, 7.06) | 17.68 | 1.88(0.99) |
| infections and infestations | hepatitis b | 9 | 3.42(1.77, 6.57) | 3.42(1.79, 6.53) | 15.31 | 1.77(0.87) |
| infections and infestations | streptococcal infection | 9 | 2.76(1.43, 5.31) | 2.76(1.45, 5.27) | 10.06 | 1.46(0.57) |
| infections and infestations | pneumonia viral | 8 | 3.51(1.75, 7.02) | 3.51(1.77, 6.97) | 14.28 | 1.81(0.86) |
| infections and infestations | subcutaneous abscess | 8 | 2.89(1.44, 5.79) | 2.89(1.46, 5.74) | 9.86 | 1.53(0.58) |
| investigations | hepatic enzyme abnormal | 8 | 2.59(1.3, 5.19) | 2.59(1.3, 5.14) | 7.82 | 1.37(0.43) |
| skin and subcutaneous tissue disorders | skin atrophy | 8 | 2.2(1.1, 4.4) | 2.2(1.11, 4.37) | 5.19 | 1.13(0.19) |
| skin and subcutaneous tissue disorders | lichen planus | 7 | 5.26(2.5, 11.05) | 5.26(2.5, 11.08) | 23.97 | 2.39(1.38) |
| injury, poisoning and procedural complications | skin injury | 7 | 3.5(1.66, 7.34) | 3.5(1.66, 7.37) | 12.42 | 1.8(0.8) |
| infections and infestations | atypical pneumonia | 7 | 3.3(1.57, 6.93) | 3.3(1.57, 6.95) | 11.16 | 1.72(0.71) |
| infections and infestations | pulmonary tuberculosis | 7 | 3.25(1.55, 6.83) | 3.25(1.54, 6.84) | 10.88 | 1.7(0.7) |
| infections and infestations | hordeolum | 7 | 2.2(1.05, 4.62) | 2.2(1.04, 4.63) | 4.56 | 1.13(0.13) |
| infections and infestations | tinea infection | 6 | 9.45(4.23, 21.13) | 9.45(4.23, 21.11) | 44.81 | 3.23(2.15) |
| infections and infestations | coccidioidomycosis | 6 | 7.46(3.34, 16.66) | 7.46(3.34, 16.66) | 33.25 | 2.89(1.81) |
| gastrointestinal disorders | diverticular perforation | 6 | 5.94(2.66, 13.25) | 5.94(2.66, 13.27) | 24.46 | 2.56(1.49) |
| neoplasms benign, malignant and unspecified (incl cysts and polyps) | malignant melanoma in situ | 6 | 5.63(2.52, 12.56) | 5.63(2.52, 12.57) | 22.67 | 2.48(1.41) |
| neoplasms benign, malignant and unspecified (incl cysts and polyps) | cutaneous t-cell lymphoma | 6 | 5.6(2.51, 12.49) | 5.59(2.5, 12.49) | 22.49 | 2.48(1.4) |
| general disorders and administration site conditions | therapeutic response delayed | 6 | 4.16(1.87, 9.28) | 4.16(1.86, 9.29) | 14.34 | 2.05(0.98) |
| neoplasms benign, malignant and unspecified (incl cysts and polyps) | invasive ductal breast carcinoma | 6 | 3.22(1.44, 7.18) | 3.22(1.44, 7.19) | 9.15 | 1.68(0.61) |
| skin and subcutaneous tissue disorders | onychomadesis | 6 | 3.18(1.43, 7.1) | 3.18(1.42, 7.1) | 8.96 | 1.67(0.6) |
| neoplasms benign, malignant and unspecified (incl cysts and polyps) | lung adenocarcinoma | 6 | 3.05(1.37, 6.79) | 3.04(1.36, 6.79) | 8.21 | 1.6(0.53) |
| neoplasms benign, malignant and unspecified (incl cysts and polyps) | b-cell lymphoma | 6 | 3.03(1.36, 6.76) | 3.03(1.36, 6.77) | 8.13 | 1.6(0.52) |
| infections and infestations | onychomycosis | 6 | 2.76(1.24, 6.15) | 2.76(1.24, 6.16) | 6.71 | 1.46(0.39) |
| injury, poisoning and procedural complications | muscle rupture | 6 | 2.55(1.14, 5.68) | 2.55(1.14, 5.7) | 5.63 | 1.35(0.28) |
| infections and infestations | arthritis bacterial | 6 | 2.45(1.1, 5.47) | 2.45(1.1, 5.47) | 5.15 | 1.29(0.22) |
| infections and infestations | gastric infection | 6 | 2.3(1.03, 5.12) | 2.3(1.03, 5.14) | 4.39 | 1.2(0.13) |
| endocrine disorders | autoimmune thyroiditis | 6 | 2.3(1.03, 5.12) | 2.3(1.03, 5.14) | 4.39 | 1.2(0.13) |
| skin and subcutaneous tissue disorders | eczema nummular | 5 | 20.1(8.28, 48.79) | 20.09(8.32, 48.53) | 88.6 | 4.3(3.13) |
| investigations | antinuclear antibody increased | 5 | 11.37(4.71, 27.49) | 11.37(4.71, 27.47) | 46.68 | 3.49(2.33) |
| musculoskeletal and connective tissue disorders | dactylitis | 5 | 10.55(4.37, 25.49) | 10.55(4.37, 25.49) | 42.68 | 3.38(2.22) |
| nervous system disorders | myelitis transverse | 5 | 6.19(2.57, 14.91) | 6.19(2.56, 14.95) | 21.59 | 2.62(1.46) |
| infections and infestations | sialoadenitis | 5 | 5.66(2.35, 13.65) | 5.66(2.34, 13.67) | 19.07 | 2.49(1.33) |
| neoplasms benign, malignant and unspecified (incl cysts and polyps) | transitional cell carcinoma | 5 | 5.63(2.33, 13.56) | 5.63(2.33, 13.6) | 18.89 | 2.48(1.33) |
| infections and infestations | papilloma viral infection | 5 | 5.1(2.12, 12.28) | 5.1(2.11, 12.32) | 16.36 | 2.34(1.18) |
| investigations | tuberculin test positive | 5 | 4.86(2.02, 11.71) | 4.86(2.01, 11.74) | 15.25 | 2.27(1.12) |
| infections and infestations | ophthalmic herpes zoster | 5 | 3.49(1.45, 8.41) | 3.49(1.44, 8.43) | 8.86 | 1.8(0.64) |
| neoplasms benign, malignant and unspecified (incl cysts and polyps) | lung neoplasm | 5 | 3.41(1.42, 8.22) | 3.41(1.41, 8.24) | 8.5 | 1.77(0.61) |
| nervous system disorders | bell's palsy | 5 | 3.3(1.37, 7.94) | 3.3(1.37, 7.97) | 7.98 | 1.72(0.56) |
| neoplasms benign, malignant and unspecified (incl cysts and polyps) | rectal cancer | 5 | 2.6(1.08, 6.25) | 2.6(1.08, 6.28) | 4.9 | 1.37(0.22) |
| general disorders and administration site conditions | application site reaction | 5 | 2.53(1.05, 6.09) | 2.53(1.05, 6.11) | 4.62 | 1.34(0.18) |
| skin and subcutaneous tissue disorders | parapsoriasis | 4 | 29.88(11.02, 80.99) | 29.87(10.99, 81.16) | 107.81 | 4.85(3.56) |
| general disorders and administration site conditions | injection site plaque | 4 | 25.77(9.53, 69.7) | 25.77(9.48, 70.02) | 92.41 | 4.65(3.36) |
| respiratory, thoracic and mediastinal disorders | tracheomalacia | 4 | 16.8(6.24, 45.19) | 16.79(6.3, 44.74) | 58.26 | 4.04(2.76) |
| neoplasms benign, malignant and unspecified (incl cysts and polyps) | renal cancer metastatic | 4 | 11.89(4.43, 31.89) | 11.89(4.46, 31.68) | 39.33 | 3.55(2.28) |
| injury, poisoning and procedural complications | drug exposure before pregnancy | 4 | 10.52(3.92, 28.19) | 10.52(3.95, 28.03) | 34.02 | 3.38(2.1) |
| neoplasms benign, malignant and unspecified (incl cysts and polyps) | rectal adenocarcinoma | 4 | 10.42(3.89, 27.93) | 10.42(3.91, 27.76) | 33.64 | 3.37(2.09) |
| psychiatric disorders | alcohol withdrawal syndrome | 4 | 9.84(3.67, 26.38) | 9.84(3.69, 26.22) | 31.41 | 3.28(2.01) |
| skin and subcutaneous tissue disorders | perioral dermatitis | 4 | 7.23(2.7, 19.34) | 7.23(2.71, 19.26) | 21.28 | 2.84(1.57) |
| infections and infestations | prostate infection | 4 | 6.95(2.6, 18.58) | 6.95(2.61, 18.52) | 20.19 | 2.79(1.51) |
| reproductive system and breast disorders | genital rash | 4 | 5.23(1.96, 13.99) | 5.23(1.96, 13.94) | 13.61 | 2.38(1.11) |
| hepatobiliary disorders | non-alcoholic steatohepatitis | 4 | 5.19(1.94, 13.88) | 5.19(1.95, 13.83) | 13.46 | 2.37(1.1) |
| infections and infestations | vulvovaginal candidiasis | 4 | 5.15(1.93, 13.75) | 5.15(1.93, 13.72) | 13.28 | 2.36(1.09) |
| cardiac disorders | dilated cardiomyopathy | 4 | 4.96(1.86, 13.25) | 4.96(1.86, 13.22) | 12.56 | 2.3(1.03) |
| general disorders and administration site conditions | breakthrough pain | 4 | 4.7(1.76, 12.56) | 4.7(1.76, 12.52) | 11.59 | 2.23(0.96) |
| ear and labyrinth disorders | sudden hearing loss | 4 | 3.74(1.4, 9.99) | 3.74(1.4, 9.97) | 8 | 1.9(0.63) |
| skin and subcutaneous tissue disorders | dermatitis psoriasiform | 4 | 3.68(1.38, 9.82) | 3.68(1.38, 9.81) | 7.76 | 1.87(0.61) |
| skin and subcutaneous tissue disorders | skin hypertrophy | 4 | 2.69(1.01, 7.17) | 2.69(1.01, 7.17) | 4.22 | 1.42(0.16) |
| infections and infestations | viral pericarditis | 3 | 35.66(11.23, 113.22) | 35.66(11.22, 113.34) | 96.96 | 5.1(3.65) |
| skin and subcutaneous tissue disorders | nail pitting | 3 | 17.58(5.6, 55.16) | 17.58(5.64, 54.79) | 45.96 | 4.11(2.68) |
| neoplasms benign, malignant and unspecified (incl cysts and polyps) | fibroadenoma of breast | 3 | 14.81(4.73, 46.37) | 14.81(4.75, 46.16) | 37.95 | 3.86(2.43) |
| neoplasms benign, malignant and unspecified (incl cysts and polyps) | angioimmunoblastic t-cell lymphoma | 3 | 13.84(4.42, 43.3) | 13.83(4.44, 43.11) | 35.14 | 3.77(2.34) |
| infections and infestations | spontaneous bacterial peritonitis | 3 | 12.41(3.97, 38.8) | 12.41(3.98, 38.68) | 31.02 | 3.61(2.19) |
| general disorders and administration site conditions | administration site reaction | 3 | 12.11(3.88, 37.87) | 12.11(3.89, 37.74) | 30.16 | 3.58(2.15) |
| neoplasms benign, malignant and unspecified (incl cysts and polyps) | nasopharyngeal cancer | 3 | 10.73(3.44, 33.51) | 10.73(3.44, 33.44) | 26.13 | 3.41(1.98) |
| injury, poisoning and procedural complications | foreign body | 3 | 7.79(2.5, 24.28) | 7.79(2.5, 24.28) | 17.59 | 2.95(1.53) |
| infections and infestations | lower respiratory tract infection viral | 3 | 7.74(2.48, 24.13) | 7.74(2.48, 24.12) | 17.45 | 2.94(1.52) |
| skin and subcutaneous tissue disorders | chronic cutaneous lupus erythematosus | 3 | 7.7(2.47, 23.99) | 7.7(2.47, 24) | 17.32 | 2.93(1.51) |
| injury, poisoning and procedural complications | colon injury | 3 | 7.47(2.4, 23.27) | 7.47(2.4, 23.28) | 16.66 | 2.89(1.47) |
| hepatobiliary disorders | hepatobiliary disease | 3 | 7.36(2.36, 22.93) | 7.36(2.36, 22.94) | 16.34 | 2.87(1.45) |
| neoplasms benign, malignant and unspecified (incl cysts and polyps) | keratoacanthoma | 3 | 7.07(2.27, 22.03) | 7.07(2.27, 22.04) | 15.51 | 2.81(1.39) |
| skin and subcutaneous tissue disorders | ingrown hair | 3 | 6.99(2.25, 21.79) | 6.99(2.24, 21.79) | 15.28 | 2.8(1.37) |
| skin and subcutaneous tissue disorders | granuloma annulare | 3 | 6.38(2.05, 19.86) | 6.38(2.05, 19.89) | 13.5 | 2.66(1.24) |
| neoplasms benign, malignant and unspecified (incl cysts and polyps) | anogenital warts | 3 | 5.49(1.76, 17.09) | 5.49(1.76, 17.11) | 10.95 | 2.45(1.03) |
| respiratory, thoracic and mediastinal disorders | pulmonary sarcoidosis | 3 | 4.6(1.48, 14.29) | 4.59(1.47, 14.31) | 8.39 | 2.19(0.77) |
| neoplasms benign, malignant and unspecified (incl cysts and polyps) | acute leukaemia | 3 | 4.07(1.31, 12.66) | 4.07(1.31, 12.69) | 6.91 | 2.02(0.6) |
| injury, poisoning and procedural complications | vaccination complication | 3 | 3.94(1.27, 12.24) | 3.94(1.26, 12.28) | 6.54 | 1.97(0.55) |
| musculoskeletal and connective tissue disorders | connective tissue disorder | 3 | 3.65(1.17, 11.34) | 3.65(1.17, 11.38) | 5.74 | 1.86(0.44) |
| musculoskeletal and connective tissue disorders | enthesopathy | 3 | 3.57(1.15, 11.08) | 3.57(1.15, 11.13) | 5.52 | 1.83(0.41) |
| nervous system disorders | cerebrovascular disorder | 3 | 3.36(1.08, 10.45) | 3.36(1.08, 10.47) | 4.96 | 1.75(0.33) |
| skin and subcutaneous tissue disorders | seborrhoeic dermatitis | 3 | 3.26(1.05, 10.14) | 3.26(1.05, 10.16) | 4.69 | 1.7(0.28) |
| gastrointestinal disorders | anorectal disorder | 3 | 3.25(1.05, 10.1) | 3.25(1.04, 10.13) | 4.66 | 1.7(0.28) |
| infections and infestations | atypical mycobacterial infection | 3 | 3.2(1.03, 9.96) | 3.2(1.03, 9.97) | 4.53 | 1.68(0.26) |

Asterisks (*) indicate novel signals not previously documented in the drug label or clinical trials. N, case number; ROR, reports odds ratio; IC, information component; IC025, the lower limit of the 95% CI of IC; CI, confidence interval.
